# Supplementary material for: Enhancing Sheep Rumen Function, and Growth Performance Through Yeast Culture and Oxalic Acid Supplementation in a Hemicellulose-Based Diet
Source: Microorganisms. 2025 Dec 12;13(12):2834. doi: 10.3390/microorganisms13122834 (PMC12735587; doi:10.3390/microorganisms13122834)
Supplement: Supplementary file 1 [file microorganisms-13-02834-s001.zip › microorganisms-3982434-supplementary.pdf]

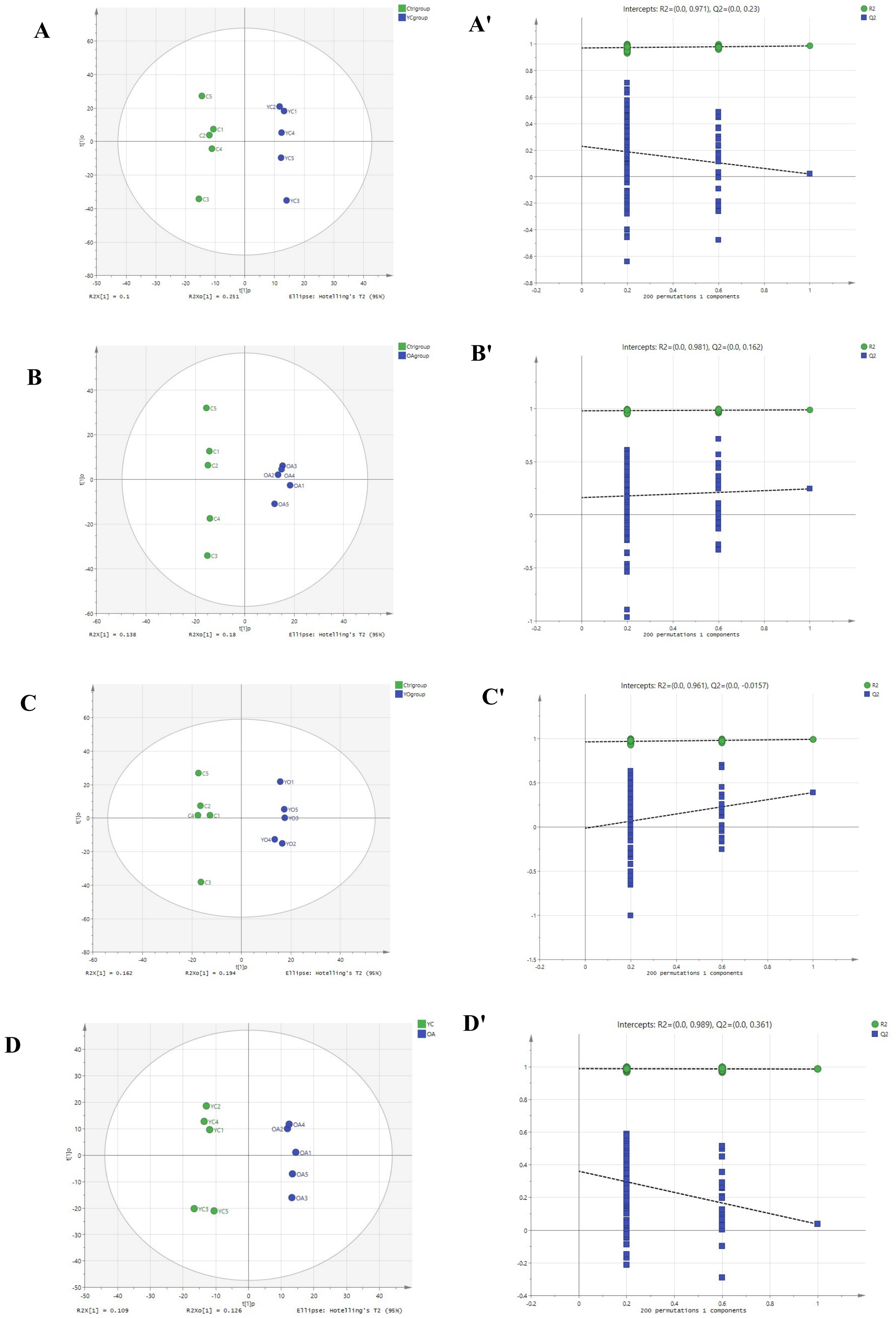
A
Ctrlgroup
YCgroup
R2X[1] = 0.1
R2Xo[1] = 0.251
Ellipse: Hotelling's T2 (95%)
A'
Intercepts: R2=(0.0, 0.971), Q2=(0.0, 0.23)
200 permutations 1 components
B
Ctrlgroup
OAgroup
R2X[1] = 0.138
R2Xo[1] = 0.18
Ellipse: Hotelling's T2 (95%)
B'
Intercepts: R2=(0.0, 0.981), Q2=(0.0, 0.162)
200 permutations 1 components
C
Ctrlgroup
YOgroup
R2X[1] = 0.162
R2Xo[1] = 0.194
Ellipse: Hotelling's T2 (95%)
C'
Intercepts: R2=(0.0, 0.961), Q2=(0.0, -0.0157)
200 permutations 1 components
D
YC
OA
R2X[1] = 0.109
R2Xo[1] = 0.126
Ellipse: Hotelling's T2 (95%)
D'
Intercepts: R2=(0.0, 0.989), Q2=(0.0, 0.361)
200 permutations 1 components

E

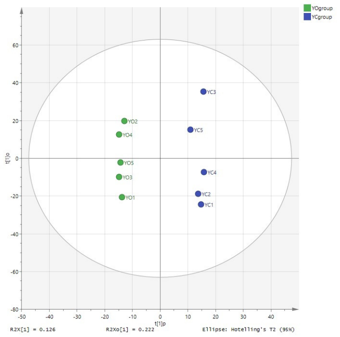

E'

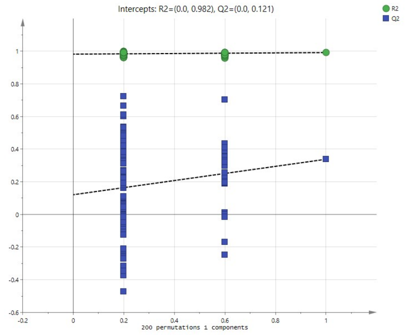

F

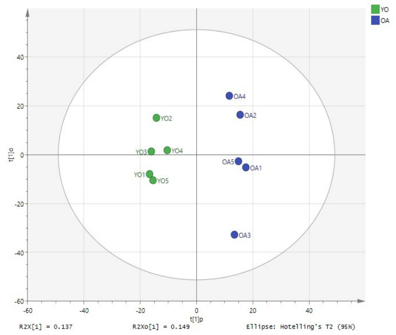

F'

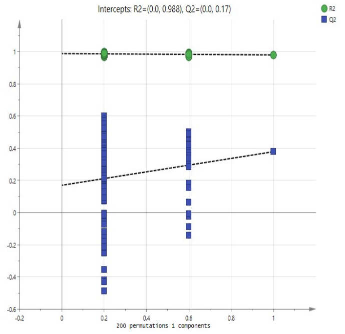

Figure S1. OPLS-DA dispersion dot plot and permutation test plot for the differential metabolites in sheep serum across different experimental groups. (A and A': OPLS-DA dispersion and permutation test plots between the ctrl and YC groups; B and B': OPLS-DA dispersion and permutation test plots between the ctrl and OA groups; C and C': OPLS-DA dispersion and permutation test plots between the ctrl and YO groups; D and D': OPLS-DA dispersion and permutation test plots between YC and OA groups; E and E': OPLS-DA dispersion and permutation test plots between the YC and YO groups; F and F': OPLS-DA dispersion and permutation test plots between the OA and YO groups, respectively).

Note: Four experimental groups with 5 replications each (n = 20) were analyzed. The graphs show predicted principal component score (t[1]p) on the x-axis and orthogonal principal component score (t[1]o) on the y-axis, with different groups distinguished by scatter shape and color. All samples fall within the 95% confidence interval (Hotelling's T-squared ellipse), indicating clear group separation. In the permutation test plot, displacement retention is on the x-axis, and R2 or Q2 values are on the y-axis. R2 is represented by a green dot, Q2 by a blue square dot, with regression lines displayed. High R2 and Q2 values close to 1 indicate a well-fitted model explaining sample differences effectively. Decreasing retention leads to lower R2 and Q2 values in the stochastic model, demonstrating model robustness without overfitting.

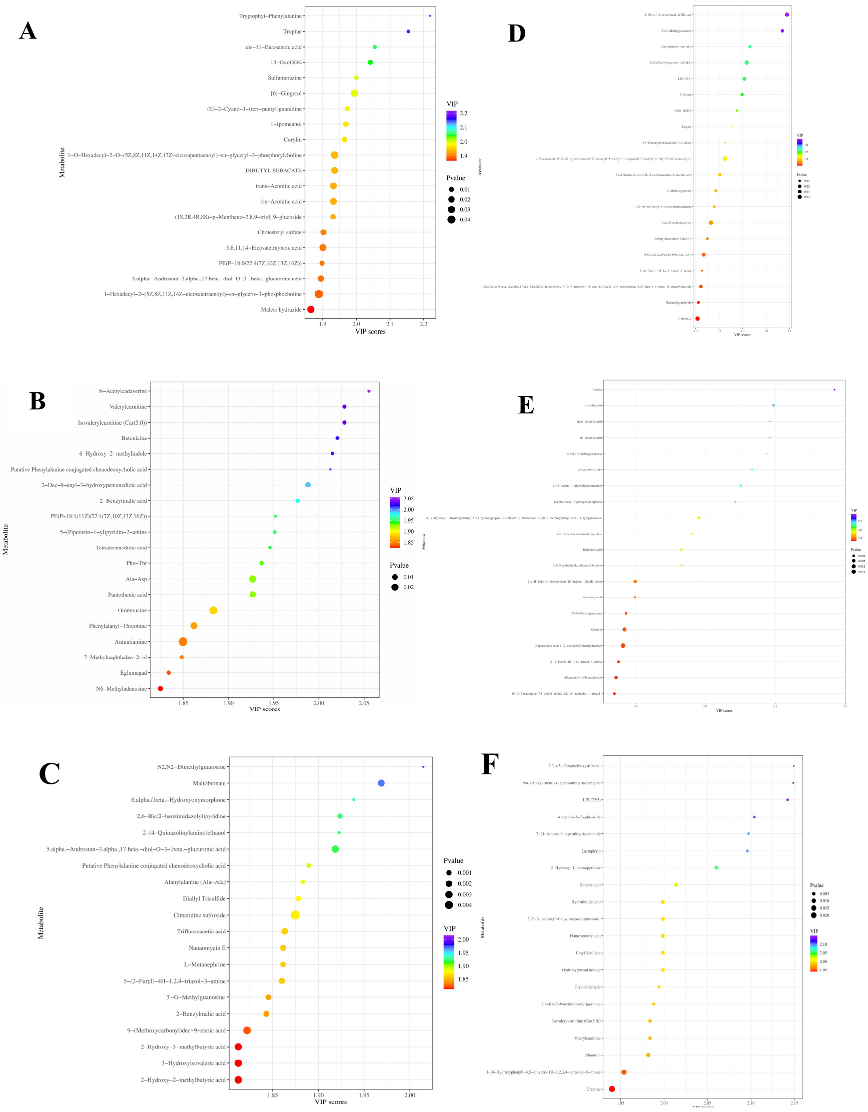

Figure S2. Bubble map showing the top 20 differential metabolites in sheep blood across different experimental groups. (A: ctrl and YC; B: ctrl and OA; C: ctrl and YO; D: YC and OA; E: YC and YO; F: OA and YO groups).

Note: We analyzed four experimental groups with five replications each (n = 20). The bubble plot displays the top 20 differential metabolites, with each dot representing a metabolite. The x-axis shows the variable projection importance score (VIP), and the y-axis displays the metabolites. Dot color indicates the VIP value, whereas dot size represents the P-value.

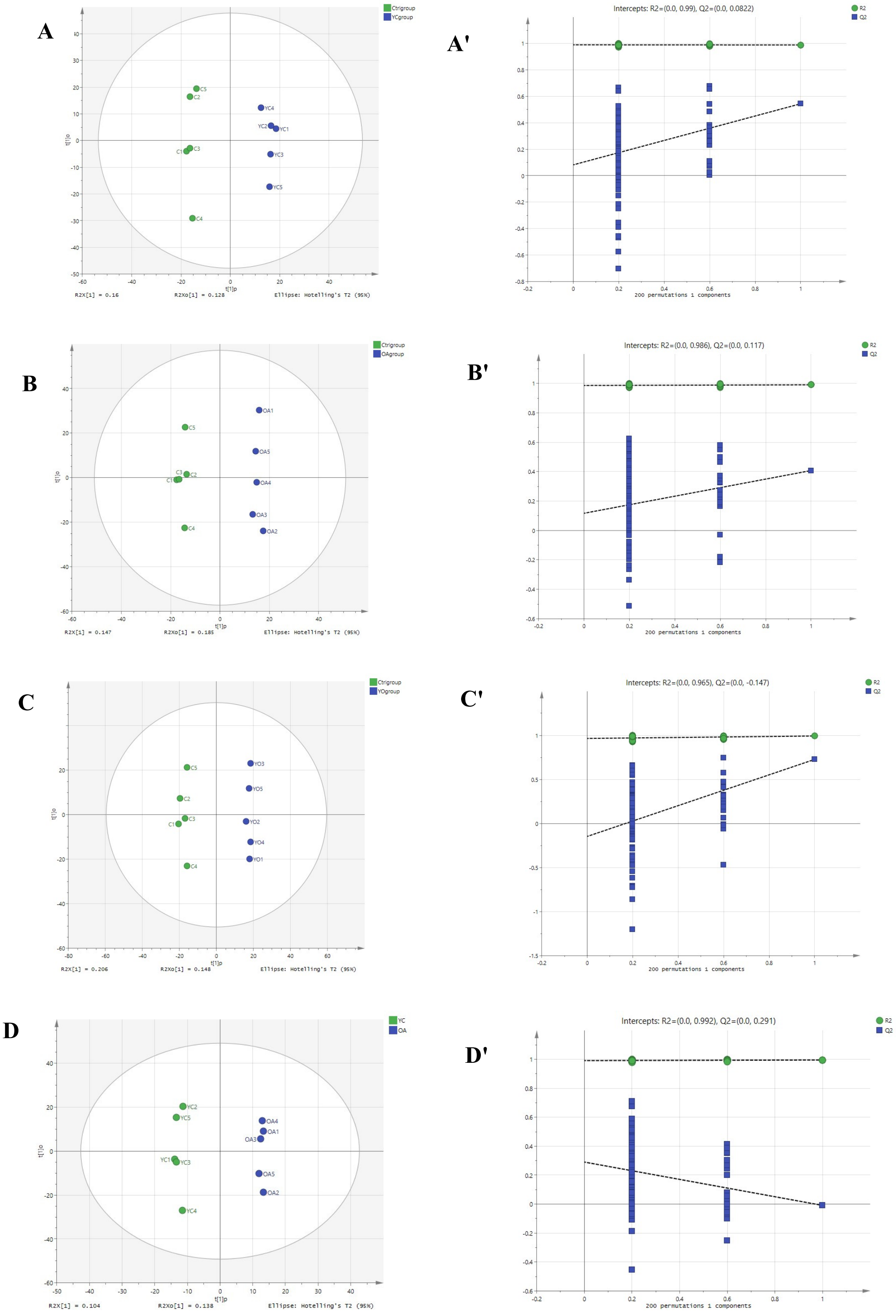
A
A'
B
B'
C
C'
D
D'
Ctrlgroup
YCgroup
OAgroup
YOgroup
YC
OA
R2
Q2
R2X[1] = 0.16
R2Xo[1] = 0.128
R2X[1] = 0.147
R2Xo[1] = 0.185
R2X[1] = 0.206
R2Xo[1] = 0.148
R2X[1] = 0.104
R2Xo[1] = 0.138
Ellipse: Hotelling's T2 (95%)
Intercepts: R2=(0.0, 0.99), Q2=(0.0, 0.0822)
Intercepts: R2=(0.0, 0.986), Q2=(0.0, 0.117)
Intercepts: R2=(0.0, 0.965), Q2=(0.0, -0.147)
Intercepts: R2=(0.0, 0.992), Q2=(0.0, 0.291)
200 permutations 1 components

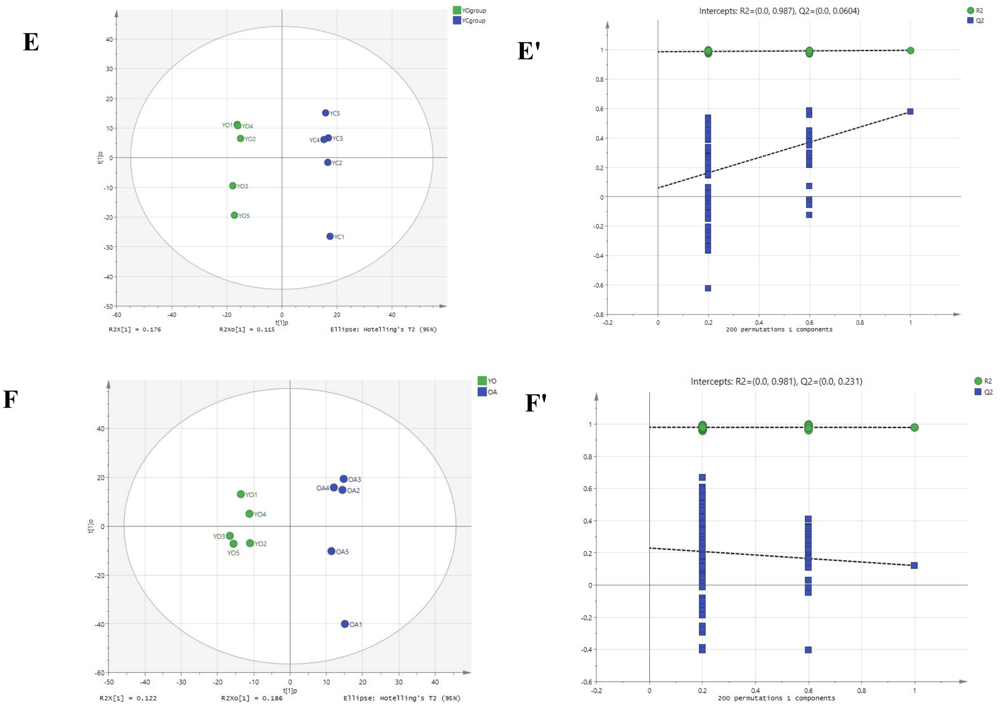

Figure S3. the OPLS-DA dispersion dot plot and permutation test plot for the differential metabolites in longissimus dorsi muscle across different experimental groups. (A and A': OPLS-DA dispersion and permutation test plots between the ctrl and YC groups; B and B': OPLS-DA dispersion and permutation test plots between the ctrl and OA groups; C and C': OPLS-DA dispersion and permutation test plots between the ctrl and YO groups; D and D': OPLS-DA dispersion and permutation test plots between YC and OA groups; E and E': OPLS-DA dispersion and permutation test plots between the YC and YO groups; F and F': OPLS-DA dispersion and permutation test plots between the OA and YO groups, respectively).

Note: Four experimental groups with 5 replications each (n = 20) were analyzed. The graphs show predicted principal component score (t[1]p) on the x-axis and orthogonal principal component score (t[1]o) on the y-axis, with different groups distinguished by scatter shape and color. All samples fall within the 95% confidence interval (Hotelling's T-squared ellipse), indicating clear group separation. In the permutation test plot, displacement retention is on the x-axis, and R2 or Q2 values are on the y-axis. R2 is represented by a green dot, Q2 by a blue square dot, with regression lines displayed. High R2 and Q2 values close to 1 indicate a well-fitted model explaining sample differences effectively. Decreasing retention leads to lower R2 and Q2 values in the stochastic model, demonstrating model robustness without overfitting.

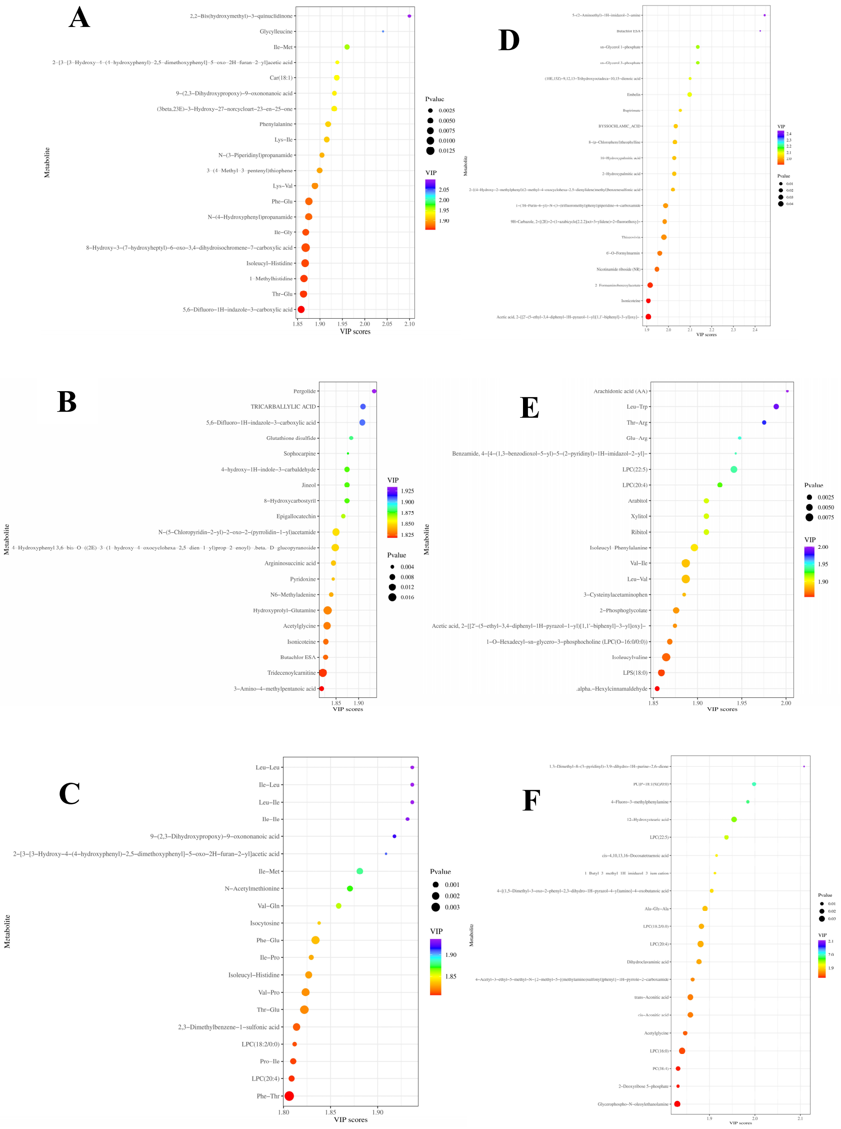

Figure S4. Bubble map showing the top 20 differential metabolites in sheep *Longissimus dorsi* muscle across different experimental groups. (A: ctrl and YC; B: ctrl and OA; C: ctrl and YO; D: YC and OA; E: YC and YO; F: OA and YO groups).

Note: We analyzed four experimental groups with five replications each (n = 20). The bubble plot displays the top 20 differential metabolites, with each dot representing a metabolite. The x-axis shows the variable projection importance score (VIP), and the y-axis displays the metabolites. Dot color indicates the VIP value, whereas dot size represents the P-value.

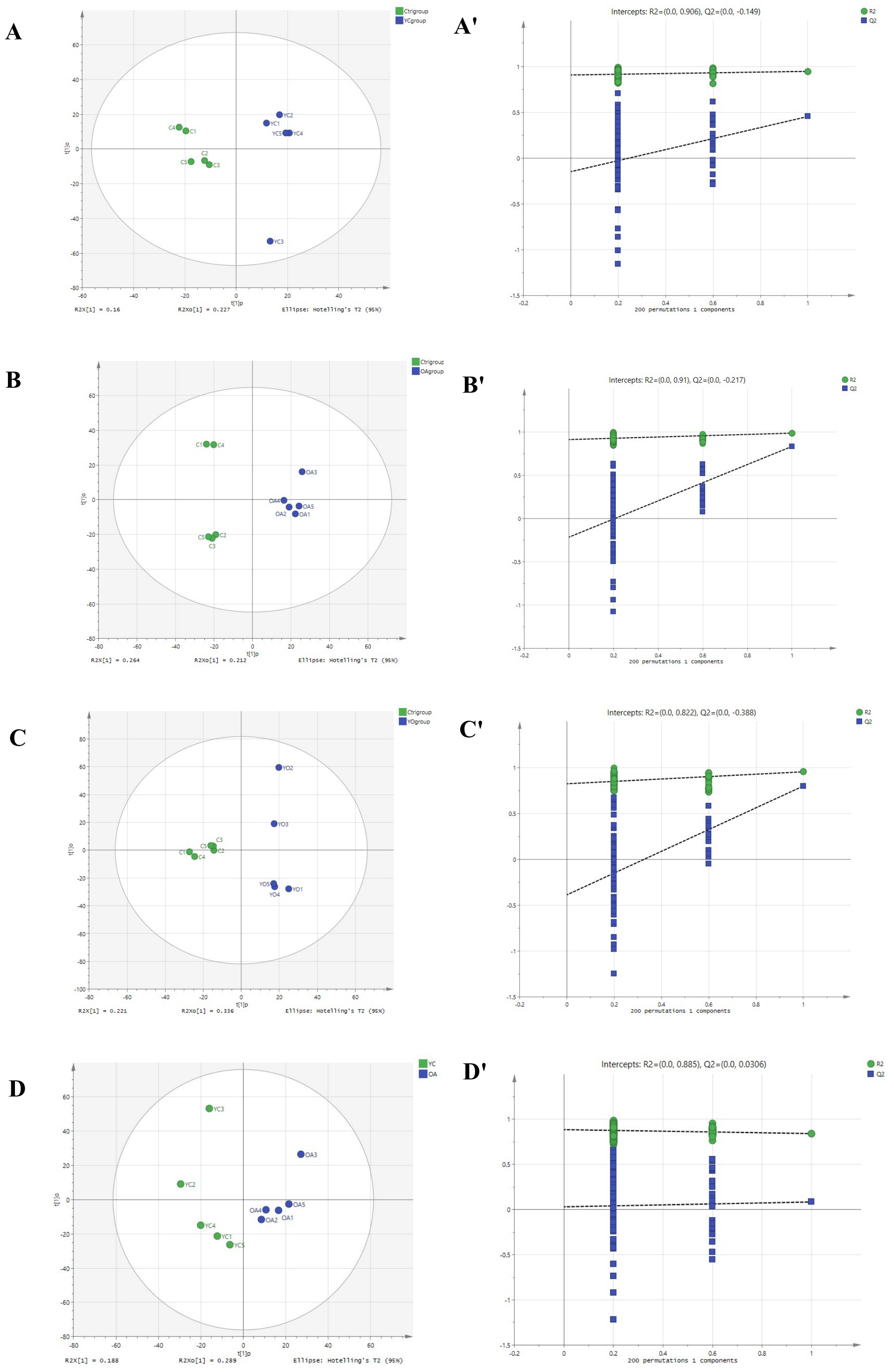
A
A'
B
B'
C
C'
D
D'
Ctrlgroup
YCgroup
OAgroup
YOgroup
YC
OA
R2
Q2
Intercepts: R2=(0.0, 0.906), Q2=(0.0, -0.149)
Intercepts: R2=(0.0, 0.91), Q2=(0.0, -0.217)
Intercepts: R2=(0.0, 0.822), Q2=(0.0, -0.388)
Intercepts: R2=(0.0, 0.885), Q2=(0.0, 0.0306)
R2X[1] = 0.16
R2Xo[1] = 0.227
R2X[1] = 0.264
R2Xo[1] = 0.212
R2X[1] = 0.221
R2Xo[1] = 0.336
R2X[1] = 0.188
R2Xo[1] = 0.289
Ellipse: Hotelling's T2 (95%)
200 permutations 1 components
t[1]p
t[1]o

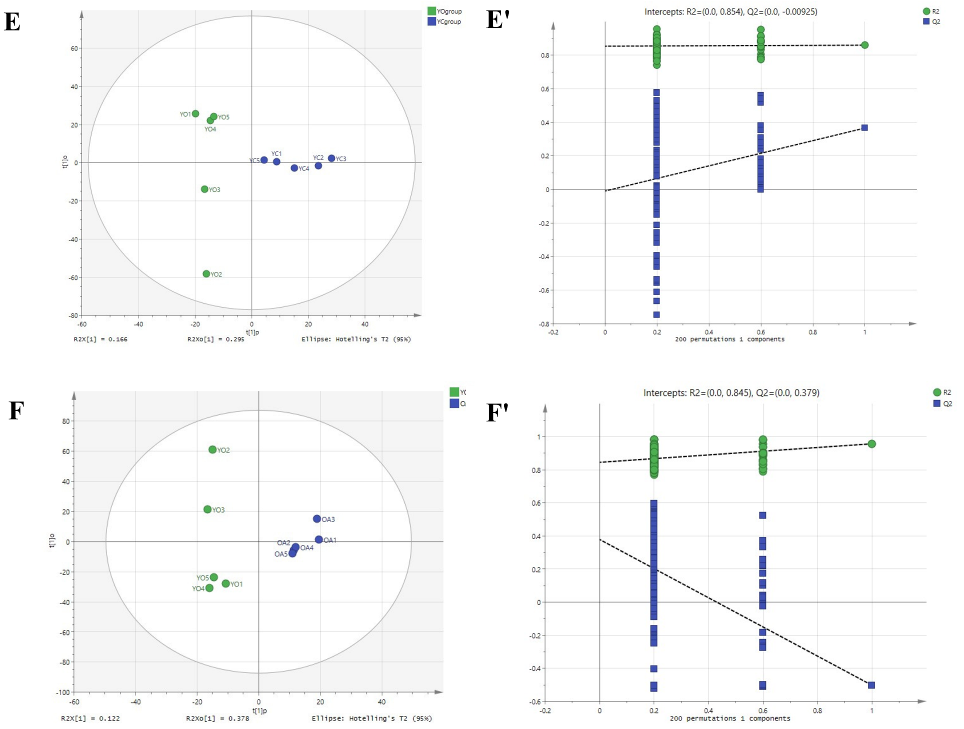

Figure S5. The OPLS-DA dispersion dot plot and permutation test plot for the differential metabolites in rumen fluid across different experimental groups. (A and A': OPLS-DA dispersion and permutation test plots between the ctrl and YC groups; B and B': OPLS-DA dispersion and permutation test plots between the ctrl and OA groups; C and C': OPLS-DA dispersion and permutation test plots between the ctrl and YO groups; D and D': OPLS-DA dispersion and permutation test plots between YC and OA groups; E and E': OPLS-DA dispersion and permutation test plots between the YC and YO groups; F and F': OPLS-DA dispersion and permutation test plots between the OA and YO groups, respectively).

Note: Four experimental groups with 5 replications each (n = 20) were analyzed. The graphs show predicted principal component score (t[1]p) on the x-axis and orthogonal principal component score (t[1]o) on the y-axis, with different groups distinguished by scatter shape and color. All samples fall within the 95% confidence interval (Hotelling's T-squared ellipse), indicating clear group separation. In the permutation test plot, displacement retention is on the x-axis, and R2 or Q2 values are on the y-axis. R2 is represented by a green dot, Q2 by a blue square dot, with regression lines displayed. High R2 and Q2 values close to 1 indicate a well-fitted model explaining sample differences effectively. Decreasing retention leads to lower R2 and Q2 values in the stochastic model, demonstrating model robustness without overfitting.

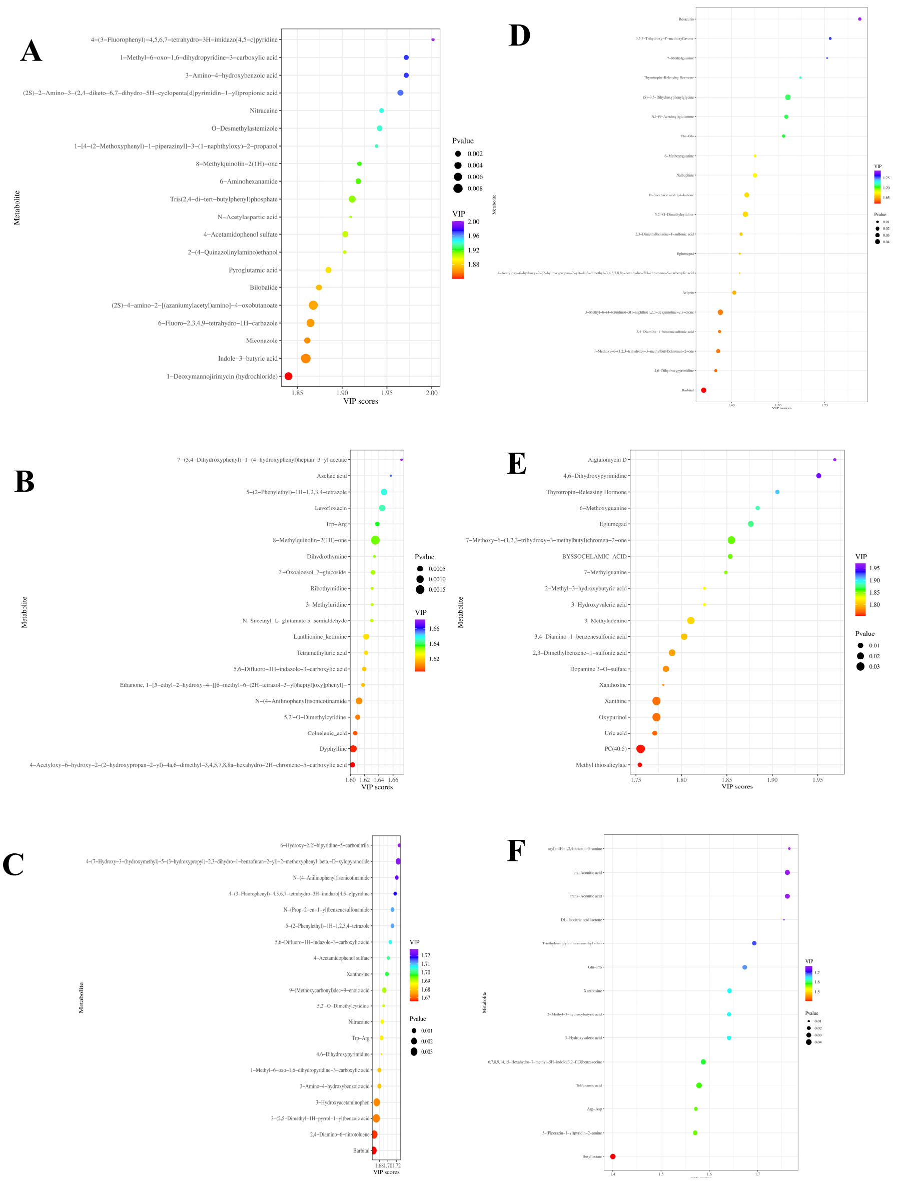

Figure S6. Bubble map showing the top 20 differential metabolites in rumen fluid across different experimental groups. (A: ctrl and YC; B: ctrl and OA; C: ctrl and YO; D: YC and OA; E: YC and YO; F: OA and YO groups). Note: We analyzed four experimental groups with five replications each (n = 20). The bubble plot displays the top 20 differential metabolites, with each dot representing a metabolite. The x-axis shows the variable projection importance score (VIP), and the y-axis displays the metabolites. Dot color indicates the VIP value, whereas dot size represents the P-value.
